# SugarBindDB, a resource of glycan-mediated host–pathogen interactions

**DOI:** 10.1093/nar/gkv1247

**Published:** 2015-11-17

**Authors:** Julien Mariethoz, Khaled Khatib, Davide Alocci, Matthew P. Campbell, Niclas G. Karlsson, Nicolle H. Packer, Elaine H. Mullen, Frederique Lisacek

**Affiliations:** 1 Proteome Informatics Group, SIB Swiss Institute of Bioinformatics, Geneva, Switzerland; 2 Glycoinformatics Inc., Great Falls, VA, USA; 3 Department of Computer Science, University of Geneva, Geneva, Switzerland; 4 Biomolecular Frontiers Research Centre, Macquarie University, North Ryde, NSW, Australia; 5 University of Gothenburg, Sahlgrenska Academy, Institute of Biomedicine, Department of Medical Biochemistry and Cell Biology, Gothenburg, Sweden; 6 The MITRE Corporation, McLean, VA, USA

## Abstract

The SugarBind Database (SugarBindDB) covers knowledge of glycan binding of human pathogen lectins and adhesins. It is a curated database; each glycan–protein binding pair is associated with at least one published reference. The core data element of SugarBindDB is a set of three inseparable components: the pathogenic agent, a lectin/adhesin and a glycan ligand. Each entity (agent, lectin or ligand) is described by a range of properties that are summarized in an entity-dedicated page. Several search, navigation and visualisation tools are implemented to investigate the functional role of glycans in pathogen binding. The database is cross-linked to protein and glycan-relaled resources such as UniProtKB and UniCarbKB. It is tightly bound to the latter via a substructure search tool that maps each ligand to full structures where it occurs. Thus, a glycan–lectin binding pair of SugarBindDB can lead to the identification of a glycan-mediated protein–protein interaction, that is, a lectin–glycoprotein interaction, via substructure search and the knowledge of site-specific glycosylation stored in UniCarbKB. SugarBindDB is accessible at: http://sugarbind.expasy.org.

## INTRODUCTION

Glycosylation is the addition of glycan/carbohydrate/oligosaccharide/sugar molecules to proteins and/or lipids. This modification enhances the functional diversity of proteins and influences their biological activities while it gives glycolipids an essential role to play in cellular recognition (1). Glycans not only modify proteins or lipids overlaying the surface of cells, but they also offer numerous binding opportunities influencing cell–cell interactions. Information on these binding events is therefore crucial to feed our understanding of intercellular communication. However, even in cases such as host–pathogen interactions, which have been extensively studied over decades, information that is recorded across diverse resources (e.g. 2,3), does not cover details of the recognition of host glycans by the pathogen proteins, known as lectins (or adhesins if the binding partner is unknown). Nonetheless, these facts have been published throughout the medical and biochemical literature since the mid-1970s (4). To bridge this gap, we introduce the SugarBind Database (SugarBindDB), a curated database developed to cover knowledge of glycan binding by human pathogen lectins.

SugarBindDB was created in 2002 within the MITRE Corporation and publicly released in 2005. It was originally designed as a complement to a pathogen-capture technology based on the binding of viral, bacterial and biotoxin lectins to specific glycans displayed on glycoprotein films. This approach relied mainly on GlycoSuiteDB (5) and UniProtKB/Swiss-Prot (6) to identify glycoproteins bearing a sugar sequence similar to an implicated glycan ligand of a pathogen lectin. In 2010, the SugarBindDB was transferred to the SIB Swiss Institute of Bioinformatics where it was integrated in the ExPASy server (7). With this transfer, SugarBindDB was added to a collection of glycoscience databases addressing the increasing bioinformatics needs created by recent technological advances in glycomics analysis (8). The database has since been co-developed within an international consortium striving to connect and integrate glycomics data with other –omics knowledgebases. To this end, these databases (9,10) share the same framework with user-friendly interfaces and are extensively cross-referenced to relevant existing bioinformatics resources. The transferred content described in (11) was gradually augmented and a new implementation matching that of sister database UniCarbKB (10) was launched in 2013. The new version introduced user-friendly data browsing and searching as first described in (12). New features and content are regularly added and released quarterly on average.

A glycan is a branched tree-like molecule synthesized from eight common monosaccharide building blocks in eukaryotes and dozens in prokaryotes (1). Details can be found in MonosaccharideDB (13). The *Essentials of Glycobiology* textbook (1) recommends a cartoon symbol for each building block and their assembly into a two-dimensional structural notation. This depiction is now widely accepted among glycobiolgists and therefore has been used in reference databases such as GlycomeDB (14) and UniCarbKB (10). It was naturally also adopted for SugarBindDB. Only part of the full glycan structure is often recognized by lectins and ‘*glycan determinant*’ or ‘*glycoepitope*’ usually designate the particular sub-structure involved directly in the binding. Over one hundred of these ligands have been characterized and are actually catalogued in the GlycoEpitope database (15) and in Glyco3D (16). They are sometimes given common names, as for instance, the glycan ligands of the well-known blood group-related ABO and Lewis antigen systems. The core data element of SugarBindDB is a set of three inseparable components: the pathogenic agent, a lectin adhesin and a glycan ligand. Each of these entities is named with as much precision as possible: taxonomic designation for pathogen agents, protein name for lectins and epitope name for ligands. Synonyms are listed whenever reported. When names are missing (which is frequent for lectins and pathogen strain names, especially in older literature), then these entities are labelled N/S meaning ‘non specified’. The database includes additional information to supplement the core data, such as related diseases and affected tissues or organs. SugarBindDB content is displayed in views. For example, an agent view will show the taxonomic name linked to the NCBI Taxonomy database, agent properties (e.g. morphology, motility, etc.), the structures of stored glycan ligands associated with this agent, and the reference(s) providing evidence for the agent–ligand relationship linked to NCBI/PubMed. Each glycan–protein binding pair is associated with at least one published reference. A view also lists a range of links connecting to further information, either internal or external to the database.

The following describes and illustrates the database content as well as the usage of search, navigation and visualisation tools that are implemented to consult or reveal information. The present version of the database accessible at: http://sugarbind.expasy.org, contains 1256 (agent, lectin, ligand) combinations backed by 174 publications.

## DATABASE OVERVIEW

### Design and implementation

SugarBindDB is built with the open-source framework Play (Release 2.2.6) written in Java and Scala, which follows the model-view-controller architecture. The views (user-interface) are predominantly written in Scala and include JQuery and Bootstrap Javascript libraries. The model and controller layers are written in Java and the Ebean object-relational mapping (ORM) library is used to query the underlying database model.

SugarBindDB uses PostgreSQL (Version 9.2) as the underlying database system that consists of multiple schemas to ensure data integrity by managing structural, literature and experimental data collections. At the time of writing the migration to PostgreSQL (Version 9.4) is being carried out.

Visualisation tools have been developed with the D3.js library (Version 3.4.12).

### Standard notation and encoding

**Figure 1.**
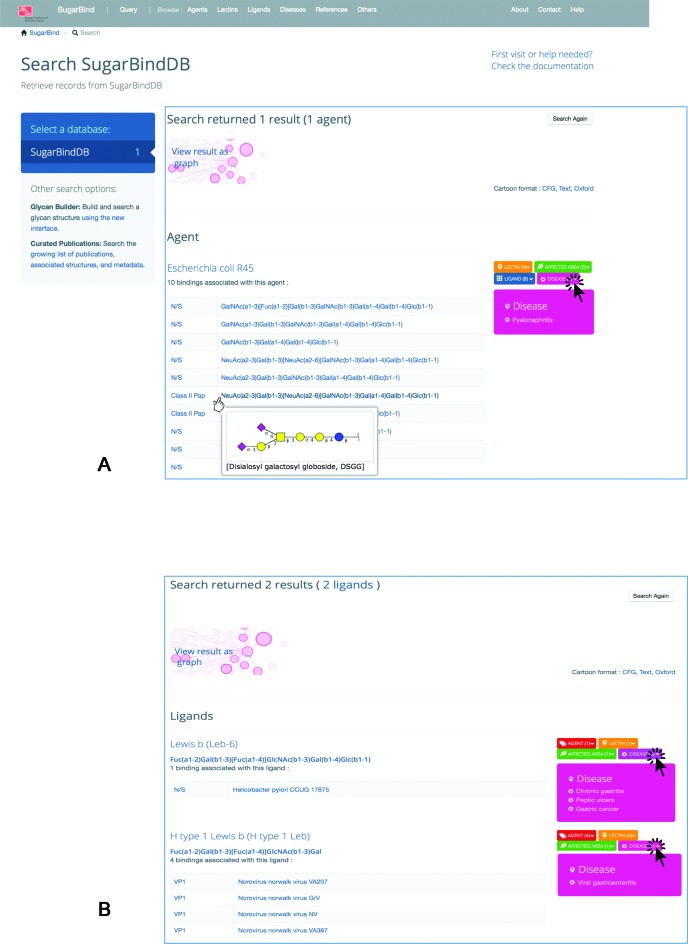
(**A**) Result page prompted by querying SugarBindDB with the pathogen/agent name ‘Escherichia coli R45’. The page is organized in blocks as a function of the query. In this case, since the query is an agent, the header is the name of the agent. Then, all bindings reported in the database for this agent are shown. The glycan molecule is presented linearly for the sake of simplicity but mousing over the formula prompts a two-dimensional cartoon presenting the Essentials of Glycobiology 2nd Edition/CFG nomenclature symbols. The association boxes are displayed on the right side with a colour code corresponding to the entity type, orange for lectins, red for agents, green for affected area/tissue, pink for disease. Clicking on any of these boxes prompts the list of entities sharing the same binding. The screen capture shows the effect of clicking on disease. (**B**) Result page prompted by querying SugarBindDB with the ligand sequence: Fuc(a1–2)Gal(b1–3)[Fuc(a1–4)]GlcNAc(b1–3)Gal. In this case, the header is the name of the ligand and two answers are returned. It is shown here that two unrelated pathogens a bacterium (H. pylori) and a virus (Norovirus Norwalk) recognize a similar glycan ligand.

The default display of glycan structures is the most commonly used notation described in the textbook *Essentials in Glycobiology* (1) and promoted by the Consortium for Functional Glycomics (CFG: http://www.functionalglycomics.org). Two other options of popular notation are the two-dimensional ‘IUPAC condensed’ and ‘Oxford’ standards (17). Clicking on ‘Cartoon format’ (see Figure 1) on the upper right hand side changes the display. The Essentials/CFG graphic representation assigns a coloured shape to each monosaccharide (e.g. yellow circle for galactose, shortened as Gal) and links components in a graph. Shared colours or shapes generally denote structural similarity among monosaccharides. For example, N-Acetylgalactosamine (yellow square) differs from galactose (yellow circle) through a so-called substituent (removal of an OH group and addition of an amino-acetyl group).

Glycans have long been encoded in the IUPAC linear format (18), that is, as regular expressions delineating branching structures with different bracket types. Such encoding can generate directional/linkage/topology ambiguity and is not sufficient in the handling of incomplete or repeated units. More recently, several encoding formats for glycans have been developed based on sets of nodes and edges, e.g. GlycoCT (19) and WURCS (20), that are suited to solving topological ambiguities and providing unequivocal descriptions. To date the GlycoCT format is acknowledged as the default format for data sharing between glycan databases (21) and consequently the most commonly used format for storing structural data.

### Data curation

The content of SugarBindDB is derived from screening the literature. This task as well as information extraction is performed by experts with selective criteria (e.g. type of method, structure resolution, etc.). Particular care was given to identifying medical information describing tissues, symptoms and diseases. Ideally controlled vocabularies and ontologies need to be adopted for easier data curation. Protein, tissue and disease description in SugarBindDB rely on UniProtKB guidelines (http://www.uniprot.org/help/controlled_vocabulary). Apart from these, the creators of SugarBindDB have opted to keep the authors’ descriptor nomenclature in each published article. This position is proving less sustainable in view of our efforts to now cross-link SugarBindDB with glycan array data and nomenclature will remain an issue until the controlled vocabularies and ontologies become more widely adopted. For this manuscript, a comprehensive literature and database screen was undertaken to provide a unified set of glycoepitopes with known synonyms. This dictionary will soon be available and its use will facilitate compliance with developing standard nomenclature.

## BROWSING AND SEARCHING

There are two options for accessing information regarding pathogens and their associated lectins: browsing or querying the database. These two approaches are available from the menu bar at the top of the homepage. Browsing is possible from the upper bar menu shaded in grey. It is of course, less targeted than querying, though in the end, the same information can be reached.

The query page is prompted from the homepage by clicking on ‘Start here with SugarBind’. Query terms belong to six predefined categories: agent, ligand, lectin, affected area, references or diseases. Auto-completion is activated upon user input. Queries may combine several terms either within the same category, for example a list of pathogen names (‘agent’ option selected), or across categories when the ‘multi-criteria’ option is selected.

Each query result page displays a set of lectin–ligand pairs. It is structured in blocks, each corresponding to either a known or an unspecified (N/S) strain of the corresponding pathogen. These blocks are grouped under entity category headers matching the categories of entities of which names were input in the query window. Figure 1A shows an example of the result of querying with ‘Escherichia coli R45’ to illustrate this point. The header in this case is the agent. The screen capture in Figure 1A shows the various actions a user can undertake to visualize more information such as mousing over ligand sequences to see a 2D representation, or clicking on further information as detailed in the next section. For example, clicking on the ‘Disease’ box pulls down a list of known diseases associated with the listed bindings. Figure 1B shows another example of query results, but this time while inputting a ligand, namely: Fuc(a1–2)Gal(b1–3)[Fuc(a1–4)]GlcNAc(b1–3)Gal. The ligand search takes names or strings of monomers. In this particular example, 2 ligands in the database match or contain the entered string, the first one in *Helicobacter pylori* and the second in *Norovirus Norwalk*. As hinted from clicking on the disease boxes, the former is known to cause gastritis and stomach cancer while the latter is an identified cause of gastroenteritis. Ligand similarity brings out tissue similarity.

### Navigation via internal and external cross-links

As described in (10), connectivity within the database supports easy navigation and potential discovery through unsuspected similarities. Coloured association boxes are ubiquitous in the display of search results and are instantiated in each page where they occur. Upon a click, these boxes show the list of entities sharing the same properties as stored in the database. As in the case illustrated in Figure 1B, these associations may point to further similarities that are not necessarily known. For example, it is not established that Fuc(a1–2)Gal(b1–3)[Fuc(a1–4)]GlcNAc(b1–3)Gal is specifically recognized by gastro-enteric pathogens but this binding is suggested by the results of the ligand search. Note that the colour code is identical throughout: red for agents, blue for ligands, orange for lectins, green for tissue/affected area and pink for disease.

Each entity (agent, lectin or ligand) is described by a range of properties that are summarized in the entity-dedicated page. These properties are displayed on the right side of the page and linked internally or to external resources. When an internal link is activated, it leads to a new page where all corresponding ligands will be shown. For instance, clicking on any of the agent properties (e.g. ‘flagellated’) will prompt the set of all stored ligands that are known to be bound by flagellated bacteria. This is a typical internal link. Lower to the right in an entity page, other internal links appear in coloured blocks that indicate a type of association (colour coded as in Figure 1) and the corresponding number of links shown in brackets.

External links are summarized and sorted by categories in Table 1. A bacterial agent is cross-linked to its corresponding summary page in HAMAP (High quality Automated and Manual Annotation of microbial Proteomes), a bacteria-oriented subproject of the UniProtKB protein annotation scheme (22). The HAMAP summary page specifies basic properties (Gram positive/negative, (an)aerobic, motility, etc.) and whether the organism was or not fully sequenced. HAMAP's coverage is completed by links to Genomes OnLine Database (GOLD) that provides equivalent information (23). The same principle is applied to viruses with cross-links to ViralZone (24). Note that ViralZone applies reciprocal cross-referencing to SugarBindDB.

**Table 1.** List of current, soon available and planned cross-references in SugarBindDB, categorized by the type of information provided by the added link

Database name	URL	Annotation type	Ref
**Current**			
PubMed	http://www.ncbi.nlm.nih.gov/pubmed	Supporting evidence of binding	-
*Agents*			
Taxonomy	http://www.ncbi.nlm.nih.gov/taxonomy	Pathogen taxonomy	-
HAMAP	http://hamap.expasy.org	Summary description of bacteria with link to possible genome sequence	(22)
GOLD	https://gold.jgi-psf.org	Summary description of bacteria with link to possible genome sequence	(23)
ViralZone	http://viralzone.expasy.org/	Summary description of viruses with link to corresponding viral proteins	(24)
*Lectins*			
UniProtKB	http://www.uniprot.org	Lectin functional annotation	(6)
Glyco3D/lectin	http://glyco3d.cermav.cnrs.fr/search.php?type = lectin	Lectin three-dimensional structure	(16)
CFG arrays	http://www.functionalglycomics.org/glycomics/publicdata/primaryscreen.jsp	Known binding patterns of lectins (not curated data)	(36)
*Ligands*			
UniCarbKB	http://unicarbkb.org/	Full structures containing ligands	(10)
**Next release**			
*Lectins*			
Pfam	http://pfam.xfam.org/	Lectin domain classification	(37)
UniRef	http://www.uniprot.org/uniref	Lectin amino acid sequence classification	(6)
GlycanBuilder	https://code.google.com/p/glycanbuilder/	Interface for building and searching glycan structure	(30)
**Prospective**			
*Agents*			
BCSDB	http://csdb.glycoscience.ru/bacterial	Known bacterial glycans possibly recognized by human lectins	(32)
PACDB	http://jcggdb.jp/search/PACDB.cgi	Pathogen–ligand binding data	Unpub
*Lectins*			
Glycosciences lab arrays	https://glycosciences.med.ic.ac.uk/data.html	Known binding patterns (curated data)	(35)
*Ligands*			
Glyco3D	http://glyco3d.cermav.cnrs.fr	3D models of ligands	(16)
Glycam	http://glycam.org	3D models of ligands	(38)
GlycoEpitope	http://www.glycoepitope.jp	Glycan epitope characterisation	(15)
GlycomeAtlas	https://rings.t.soka.ac.jp/GlycomeAtlasV3/GUI.html	Tissue expression	(33)

Publications reporting glycan binding very seldom provide a protein name, let alone a sequence accession number for a lectin. Lectins are also very poorly annotated in bacterial genomes and in UniProtKB, while dedicated databases contain sparse information due to limited experimental data (25). That is why we are currently devoting efforts to include more protein sequence-based information that can be derived from mining the literature as well as databases. Even though this will only provide rough annotation, it may provide some leads for further investigation. It will also feed our procedure for inferring lectin names by similarity.

Finally, we are gradually including glycan structure array interaction cross-links to enhance the knowledge of binding to specific glycoepitopes. Published glycan array work such as (26) is also stored in the databases of the CFG.

### Interactive graphs

Lectin–ligand pairs associated with distinct pathogens can be visualized all together by clicking on the ‘view result as graph’ box located above a result list (see Figure 1). A new window/tab is automatically opened, showing, first, a so-called Sankey graph, which maps all information on a hierarchical graph defined in the following order: agent–lectin–ligand. The same data are plotted in Directed force graph below the Sankey graph. Each agent name (pathogen) is an initial node linked to its associated strains. Each strain is a node linked to its associated lectin(s) and each lectin is another node linked to the glycan structure that it binds. Each ligand is a final node. The links are visualized as grey connections. Clicking on any node highlights the path that goes through it via a colour change to orange. Note that clicking on any name in the graphs leads directly to the corresponding entity page.

**Figure 2.**
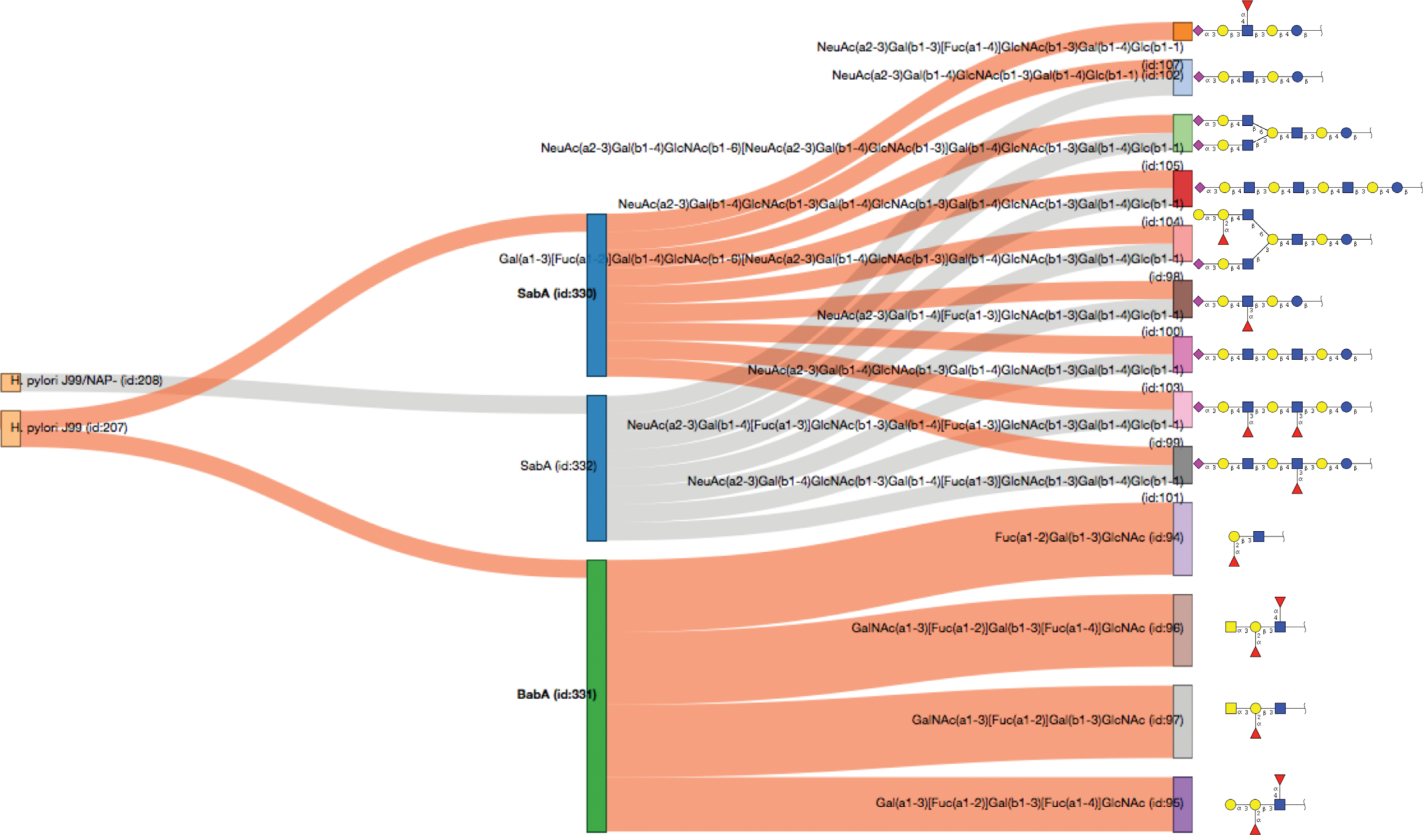
This Sankey graph highlights the specificity of the two lectins of Helicobacter pylori strain J99 in binding a variety of glycan determinants as stored in the database. Clicking on these lectins SabA and BabA in the graph changes the path colour from grey to orange. Then the specificity of each lectin is brought out by the absence of common path leading from the lectin to the glycan ligand. Moreover, the glycans that are recognized by each lectin make two distinct structural groups.

Figure 2 shows the Sankey graph for two strains of *Helicobacter pylori* and the paths (highlighted in orange) linking the two well-documented lectins BabA and SabA to their glycan ligands. The graph convincingly shows the specificity of the two lectins in recognising two distinct types of glycan molecules. While an obvious preference for O-glycoepitopes characterizes BabA binding, SabA selectively binds extended oligosaccharides attached to lipids. These observations need careful scrutiny, since the glycoconjugate type (protein or lipid) may reflect both limits in the state-of-the-art binding assay techniques as well as in our knowledge of glycan expression. With the SugarBindDB interactive graphic view, the user can investigate lectin or ligand specificity, or alternatively, compare the behaviour of different pathogens that infect the same tissues and generate similar clinical symptoms.

### Substructure search

The recent introduction of semantic web technologies in glycobioinformatics (27) has led us to rely on the standardized Resource Description Framework (RDF) format for efficient structure matching. In fact, matching glycoepitope ligands to full structures containing these substructures is the most natural link between SugarBindDB and UniCarbKB. We thus implemented a substructure search, the result of which is directly accessible from ligand pages.

In a graph representation of a glycan, each monosaccharide residue is a node possibly associated with a list of properties and each linkage is an edge also potentially associated with a list of properties. An RDF ontology that translates a glycan structure with all potential biological properties into a list of triples is described in (28). The proposed ontology is based on GlycoCT. All monosaccharides and substituents are treated as separate components and annotated in the residue list with a specific ID. The connectivity list contains the linkages between the components annotated in the residue list. Our ontology follows the same principle: all substituents are treated as separate components as opposed to merging them with their associated monosaccharides in order to avoid contaminating the model with biological assumptions. Following this ontology, glycan substructures are translated into a SPARQL query. A native support to this query language is provided by each RDF triple store, thereby not tying our solution to any particular product; however, the queries support SPARQL 1.1. Sesame API (29) together with the Java driver provided by Openlink that has been used for querying Virtuoso RDF triple store.

Structure matching is pre-calculated and included in the database. Each match is linked to the corresponding UniCarbKB glycan structure entries.

### Protein–protein interactions mediated by glycans

**Figure 3.**
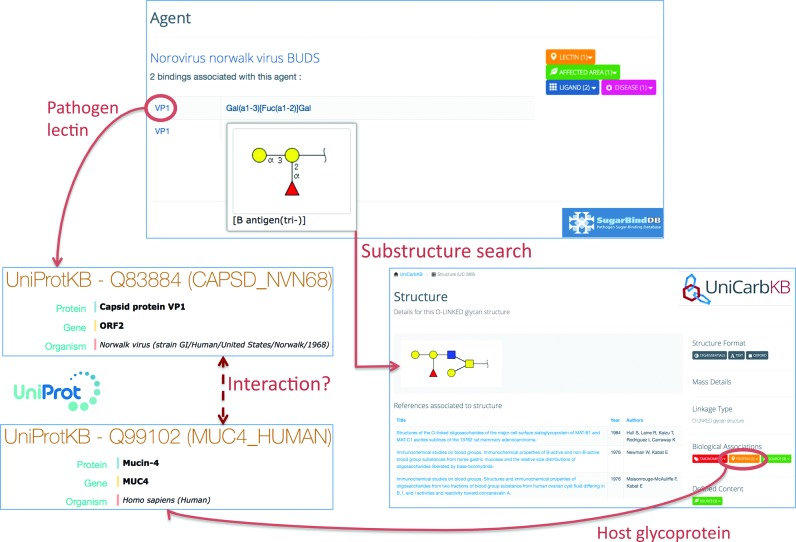
This figure shows how protein interacting partners can be found by following SugarBindDB cross-links. VP1, a viral lectin of the Norovirus Norwalk strain binds the *B antigen (tri-)* glycan determinant. The VP1 protein is linked to UniProtKB Q83884. The substructure search associates the *B antigen (tri-)* glycan determinant with 34 full glycan structure entries of UniCarbKB. One of these 34 matches is shown as an example. This structure is linked to UniProtKB Q99102 describing the host glycoprotein (MUC-4) on which the glycan is attached. The dashed line suggests a possible interaction between VP1 and MUC-4.

SugarBindDB can be used to identify protein partners interacting via glycans. This is illustrated in Figure 3. In this example, VP1, a viral lectin of the Norovirus Norwalk, is recorded as binding the so-called *B antigen (tri-)* glycan determinant/glycoepitope. The VP1 protein is linked to UniProtKB information and details of its glycan determinant can be viewed in the corresponding ligand page of SugarBindDB. In this page, the precalculated substructure search reports 34 matches of reported glycan structures that contain this determinant in UniCarbKB. Examining the corresponding full glycan structure entries shows that the vast majority of these structures are borne by mucins. Figure 3 shows one such entry and the link to the associated glycoprotein (MUC-4). With this association, it can then be hypothesized that the VP1 viral lectin interacts with a human mucin via its glycosylation. Needless to say, it is one potential interaction among many other possible ones. The careful examination of the alternative 33 structures, even though similar through a shared relation to mucins, is imperative prior to establishing the interaction as a fact.

This example emphasizes a simple and hypothesis-driven procedure that can be followed to identify protein–protein interactions mediated by glycans in four steps:

1. Start with a SugarBindDB glycan–protein binding pair and recall the UniProtKB accession number of the corresponding lectin;
2. Map the corresponding ligand to full glycan structures of UniCarbKB (via the pre-calculated sub-structure search);
3. Use cross-links of selected glycan structures to UniProtKB to retrieve information on carrier glycoproteins;
4. Correlate UniProtKB accession numbers of steps 1 and 3 to hypothesize interactions between pathogen lectin and host glycoprotein.

The initial glycan–protein binding pair of SugarBindDB is the essential piece of information to start with in order to identify potentially new protein–protein interactions.

## FUTURE PROSPECTS

One of the next features of SugarBindDB will be the integration of the Glycan Builder tool (30) that provides an interface for creating a glycan structure with the CFG/Essentials of Glycobiology symbol notation. Once drawn by the user, the 2D representation will be directly used for querying. In addition, we plan to increase the connectivity of the database as highlighted in Table 1 (prospective). We will interconnect further with the Glyco3D databases and related tools to better visualize 3D interactions (16,31). We also foresee the importance of combining the current information in SugarBindDB with the potential host response to infection via the human lectin recognition of bacterial glycans as recorded in BCSDB (32). Furthermore, glycan tissue profiling is likely to help decipher the role of glycans; and we are planning integration with GlycomeAtlas data (33). In fact, the latter two cited resources follow among others, the current coordinated move towards RDF-based data integration that is already shaping future developments of glycoscience databases (21,34). Within our consortium, UniCarbKB represents data in RDF and the RDF scheme of SugarBindDB is in preparation. In parallel, the tight integration of all resources of the Japan Consortium for Glycobiology and Glycotechnology databases (JCGGDB) relying on GlycoRDF grants access to a wealth of information. Collaboration is ongoing to build appropriate SPARQL queries and achieve our goal of boosting connectivity. In particular, GlycoEpitope or PACBD, the closest resources to SugarBindDB (though not including specific lectin information) will be our first targets for cross-linking. Last but not least, we plan to enhance the annotation of lectins by substantially increasing the number of references to glycan array experiments from the CFG as well as from the Glycosciences Lab of the Imperial College in London (35). These two sources are particularly rich for characterising viral lectins.
